# Critical Role of Bcr1-Dependent Adhesins in C. albicans Biofilm Formation In Vitro and In Vivo

**DOI:** 10.1371/journal.ppat.0020063

**Published:** 2006-07-07

**Authors:** Clarissa J Nobile, David R Andes, Jeniel E Nett, Frank J Smith, Fu Yue, Quynh-Trang Phan, John E Edwards, Scott G Filler, Aaron P Mitchell

**Affiliations:** 1 Department of Microbiology, Columbia University, New York, New York, United States of America; 2 Biological Sciences Program, Department of Biological Sciences, Columbia University, New York, New York, United States of America; 3 Department of Medicine, Section of Infectious Diseases, University of Wisconsin, Madison, Wisconsin, United States of America; 4 Los Angeles Biomedical Research Institute at Harbor-UCLA Medical Center, Torrance, California, United States of America; 5 The David Geffen School of Medicine, University of California Los Angeles, Los Angeles, California, United States of America; University of California San Francisco, United States of America

## Abstract

The fungal pathogen Candida albicans is frequently associated with catheter-based infections because of its ability to form resilient biofilms. Prior studies have shown that the transcription factor Bcr1 governs biofilm formation in an in vitro catheter model. However, the mechanistic role of the Bcr1 pathway and its relationship to biofilm formation in vivo are unknown. Our studies of biofilm formation in vitro indicate that the surface protein Als3, a known adhesin, is a key target under Bcr1 control. We show that an *als3/als3* mutant is biofilm-defective in vitro, and that *ALS3* overexpression rescues the biofilm defect of the *bcr1/bcr1* mutant. We extend these findings with an in vivo venous catheter model. The *bcr1/bcr1* mutant is unable to populate the catheter surface, though its virulence suggests that it has no growth defect in vivo. *ALS3* overexpression rescues the *bcr1/bcr1* biofilm defect in vivo, thus arguing that Als3 is a pivotal Bcr1 target in this setting. Surprisingly, the *als3/als3* mutant forms a biofilm in vivo, and we suggest that additional Bcr1 targets compensate for the Als3 defect in vivo. Indeed, overexpression of Bcr1 targets *ALS1, ECE1,* and *HWP1* partially restores biofilm formation in a *bcr1/bcr1* mutant background in vitro, though these genes are not required for biofilm formation in vitro. Our findings demonstrate that the Bcr1 pathway functions in vivo to promote biofilm formation, and that Als3-mediated adherence is a fundamental property under Bcr1 control. Known adhesins Als1 and Hwp1 also contribute to biofilm formation, as does the novel protein Ece1.

## Synopsis

The formation of biofilms (surface-attached microbial communities) on implanted medical devices such as catheters is a major cause of fungal and bacterial infections. Prior studies of the fungal pathogen Candida albicans have shown that the regulator Bcr1 is required for biofilm formation in vitro, but the mechanism through which it promotes biofilm formation and its significance for biofilm formation in vivo was uncertain. The authors demonstrate that Bcr1 is required for biofilm formation in vivo in a rat model of catheter-based infection. Manipulation of Bcr1 target genes through mutation and gene overexpression shows that the known surface adhesin Als3 has a pivotal role in biofilm formation and that adhesins Als1 and Hwp1 also contribute to biofilm formation. The results thus indicate that adherence is the key property regulated by Bcr1 and highlight a group of adhesins as potential therapeutic targets.

## Introduction

Biofilms are microbial communities that are associated with solid surfaces. Most bacteria and fungi exist predominantly in such communities in nature, and they form the basis for numerous interactions that affect human health. Cells in a biofilm display phenotypes that are distinct from their free-living counterparts, including extreme resistance to many antimicrobial agents [1–4]. Their health impact is reflected in the fact that implanted medical devices, such as intravascular catheters, are major risk factors for bloodstream and deep tissue infection [5, 6]. These devices serve as substrates for biofilm development; the mass and intrinsic drug resistance of the biofilm limits efficacy of host defenses and antimicrobial therapy. These biofilm-based infections are estimated to cause about 50% of all nosocomial infections [5, 7].

The fungal pathogen Candida albicans is a major cause of device-associated infections [5, 8, 9]. It produces adherent biofilms on a variety of different surfaces in vitro [3, 4, 10, 11]. Biofilm formation begins with surface adherence of yeast-form cells, which grow to yield a basal layer. Basal layer cells include some hyphae, or long tubular chains of cells, which extend to yield an upper layer that is almost exclusively hyphae. As the biofilm matures, it produces an extracellular matrix containing predominantly carbohydrate and protein [1, 12, 13].

C. albicans Bcr1, a C_2_H_2_ zinc finger protein, has a significant role in biofilm formation: *bcr1/bcr1* insertion and deletion mutants form only rudimentary biofilms on silicone catheter material in vitro [14]. Bcr1 is required for expression of several cell wall protein genes, and we have proposed that Bcr1 is a positive regulator of adherence. Many Bcr1 target genes had been identified initially as hyphal-specific genes, and *BCR1* RNA accumulation depends upon the hyphal developmental activator Tec1 [14]. Bcr1 is not required for hyphal morphogenesis, and we believe that it acts downstream of Tec1 to activate the acquisition of hyphal adherence properties.

Biofilms are considerably more complex in vivo than in vitro. For example, in vivo, biofilms form on intravascular catheters under conditions of vascular flow, and are exposed to and incorporate many plasma constituents. The complexity involved in forming a biofilm in vivo underscores the question of whether the same mechanisms are required for biofilm formation in vitro as in vivo. Indeed, several fungal and bacterial mutants have medium-dependent biofilm defects in vitro [15, 16]. Thus, the functions of key regulators must be appraised in vivo in order to connect questions in developmental biology to answers in antimicrobial therapy.

Recently developed animal models permit analysis of C. albicans biofilm formation in vivo. Central venous catheter infection models have been described for both rabbits [17] and rats [18]. These catheter surfaces are substrates for extensive biofilm formation, and biofilm cells on these substrates exhibit reduced antifungal susceptibility. These models further reflect the circumstances of human infection, in that the biofilm cells can lead to seeding and infection of organs [18].

In this report, we test the roles of Bcr1 target genes in biofilm formation in vitro. Our findings substantiate the proposal that Bcr1 is a regulator of adherence. We extend this analysis to an in vivo model, where our findings argue that adherence is a fundamental property under Bcr1 control that promotes biofilm formation and that the adhesin Als3 is a pivotal functional target of Bcr1 both in vitro and in vivo. Our findings highlight the complexity of in vivo biofilm formation, yet reveal a convergence of in vitro and in vivo studies to define a significant biofilm regulatory mechanism.

## Results

### Bcr1 Promotes Adherence In Vitro

**Figure 1:**
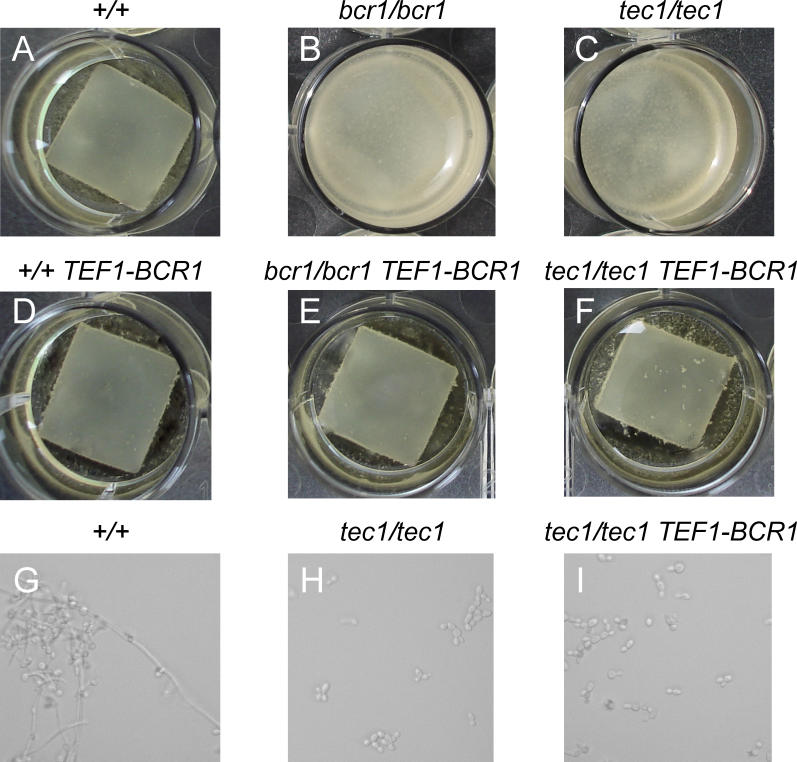
Effect of Increased *BCR1* Expression on Adherence and Hyphal Morphogenesis Strains were grown under in vitro biofilm assay conditions for 60 h and photographed (A–F) or grown in Spider suspension cultures and examined by phase contrast microscopy at ×400 magnification (G–I). For the biofilms assays, turbid medium with all cells free-floating in the medium rather than attached to the silicone substrate indicates a biofilm-negative phenotype; clear medium with the silicone substrate completely covered with cells indicates a biofilm-positive phenotype. Relevant genotypes are given above each panel for strains CJN1015 (reference strain + *TEF1*) (A, G), CJN1060 (*bcr1/bcr1* + *TEF1*) (B), CJN1052 (*tec1/tec1 + TEF1*) (C, H), CJN1039 (reference strain + *TEF1-BCR1*) (D), CJN1011 (*bcr1/bcr1* + *TEF1-BCR1*) (E), and CJN1035 (*tec1/tec1* + *TEF1-BCR1*) (F, I).

**Figure 2:**
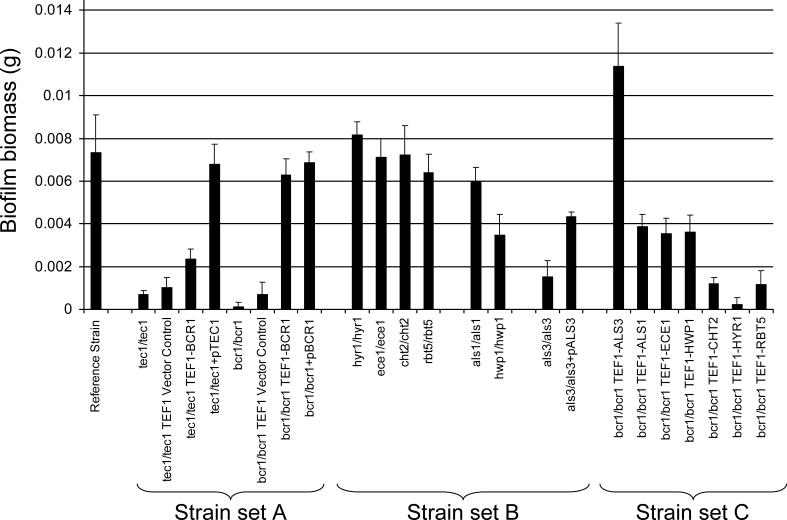
Biofilm Dry Mass Determinations Biofilm dry mass determinations were made in quadruplicate after 60 h growth under standard biofilm conditions, as detailed in Materials and Methods. Reference strains DAY185 (shown) and CAI4-URA3 (not shown) gave similar results. Strains are grouped for convenience of comparison. Strain Set A contains CJN896 (*tec1/tec1*), CJN1052 (*tec1/tec1 + TEF1*), CJN1035 (*tec1/tec1* + *TEF1-BCR1*), CJN1023 (*tec1/tec1* + p*TEC1*), CJN702 (*bcr1/bcr1*), CJN1060 (*bcr1/bcr1* + *TEF1*), CJN1011 (*bcr1/bcr1* + *TEF1-BCR1*), CJN698 (*bcr1/bcr1* + p*BCR1*), respectively. Strain Set B contains FJS2 (*hyr1/hyr1*), FJS6 (*ece1/ece1*), FJS5 (*cht2/cht2*), FJS8 (*rbt5/rbt5*), CAYC2YF1U (*als1/als1*), CAH7-1A1E2 (*hwp1/hwp1*), CAYF178U (*als3/als3*), CAQTP178U (*als3/als3* + p*ALS3*), respectively. Strain Set C contains CJN1153 (*bcr1/bcr1* + *TEF1-ALS3*), CJN1144 (*bcr1/bcr1* + *TEF1-ALS1*), CJN1288 (*bcr1/bcr1* + *TEF1-ECE1*), CJN1222 (*bcr1/bcr1* + *TEF1-HWP1*), CJN1281 (*bcr1/bcr1* + *TEF1-CHT2*), CJN1259 (*bcr1/bcr1* + *TEF1-HYR1*), CJN1276 (*bcr1/bcr1* + *TEF1-RBT5*), respectively.

We proposed that Bcr1 acts in the hyphal development pathway to promote adherence through stimulation of expression of several cell surface protein genes. This hypothesis predicts that overexpression of *BCR1* in yeast form cells may stimulate adherence. We tested this prediction by examining the effects of *BCR1* expression in a *tec1/tec1* mutant (Figures 1 and 2), which is defective in producing hyphae in vitro [19]. The *tec1/tec1* mutant is defective in biofilm formation [14], and the mutant cells fail to adhere to silicone catheter material (Figures 1A, 1C, and 2, Strain Set A). Introduction of a *TEF1-BCR1* overexpression construct restored biofilm formation ability partially to the *tec1/tec1* mutant (Figures 1F and 2, Strain Set A; *p* = 0.018 for the comparison of biomass determinations). Overexpression of *BCR1* in the *tec1/tec1* mutant does not restore hyphal formation ability (Figure 1G–1I). We believe that the partial suppression by *TEF1-BCR1* reflects the failure to restore hyphal formation to the *tec1/tec1* mutant. In any case, these findings support the idea that Bcr1 is a positive regulator of adherence, but not of hyphal formation.

**Figure 3:**
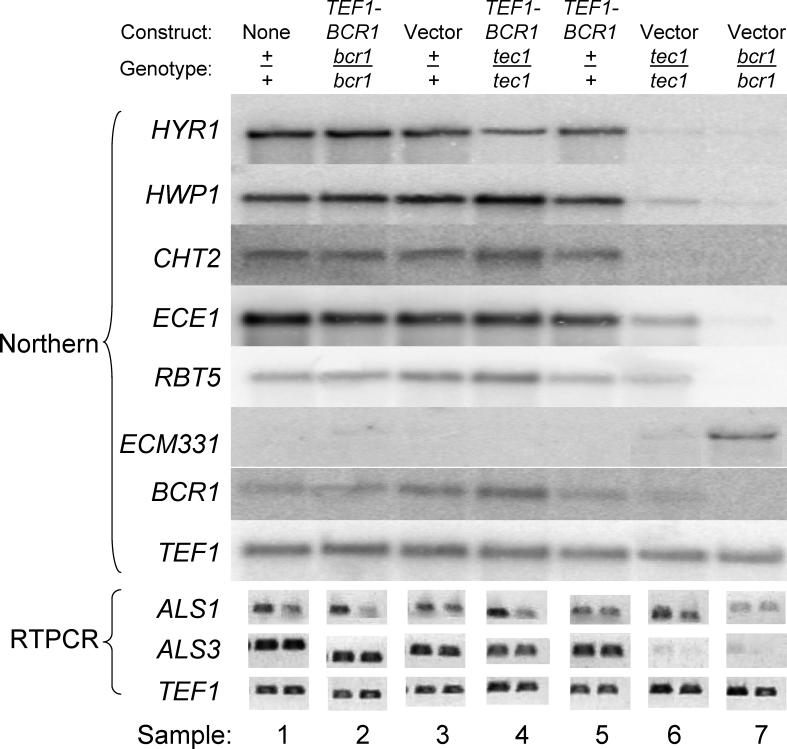
Effect of Increased *BCR1* Expression on Target Gene RNA Levels RNA prepared from mid-log phase Spider medium cultures was used to prepare Northern blots or in RT-PCR assays, as indicated. Northern blots were probed for the transcripts indicated along the left side, and PhosphorImager exposures are shown. RT-PCR assays for *ALS1, ALS3,* and *TEF1* were conducted on serial 2-fold dilutions of cDNA preparations and fractionated on agarose gels; only the last two dilutions are shown. *TEF1* transcript levels were used as an expression control. Strains included DAY185 (reference strain) (sample 1), CJN1011 (*bcr1/bcr1* + *TEF1-BCR1*) (sample 2), CJN1015 (reference strain + *TEF1*) (sample 3), CJN1035 (*tec1/tec1* + *TEF1-BCR1*) (sample 4), CJN1039 (reference strain + *TEF1-BCR1*) (sample 5), CJN1052 (*tec1/tec1 + TEF1*) (sample 6), and CJN1060 (*bcr1/bcr1* + *TEF1*) (sample 7).

To understand the mechanism of Bcr1-promoted adherence, we compared expression of Bcr1-dependent genes in strains with or without the *TEF1-BCR1* construct (Figure 3). The genes *HYR1, HWP1, CHT2, ECE1, RBT5, ALS1,* and *ALS3* were expressed at much lower levels in a *bcr1/bcr1* mutant than in the wild-type reference strain (Figure 3, samples 3 and 7). The presence of the *TEF1-BCR1* construct restored expression of these genes and biofilm formation in the *bcr1/bcr1* mutant, thus verifying the function of the construct (Figure 2; Figure 3, samples 2 and 7). The surface protein gene *ECM331* was expressed at higher levels in the *bcr1/bcr1* mutant than in the wild-type reference strain, and this elevated expression was also reversed by the *TEF1-BCR1* construct (Figure 3, samples 2, 3, and 7). We note that *TEF1-BCR1* did not substantially increase expression of Bcr1-dependent genes in the otherwise wild-type reference strain background (Figure 3, samples 5 and 3). Among the Bcr1-dependent genes, we found that *HYR1, HWP1, CHT2, ECE1,* and *ALS3* were expressed at reduced levels in the *tec1/tec1* strain compared to the reference strain (Figure 3, samples 3 and 6). Introduction of the *TEF1-BCR1* construct increased expression of these genes in the *tec1/tec1* strain (Figure 3, samples 4 and 6). These findings suggest that Bcr1 acts downstream of Tec1 to activate expression of target genes *HYR1, HWP1, CHT2, ECE1,* and *ALS3*. Furthermore, the augmented adherence during biofilm formation of the *tec1/tec1 TEF1-BCR1* strain highlights this particular group of Bcr1-dependent genes as candidates for mediators of Bcr1-dependent adherence.

### Key Role of Bcr1 Target Gene *ALS3* in Biofilm Formation In Vitro

**Figure 4:**
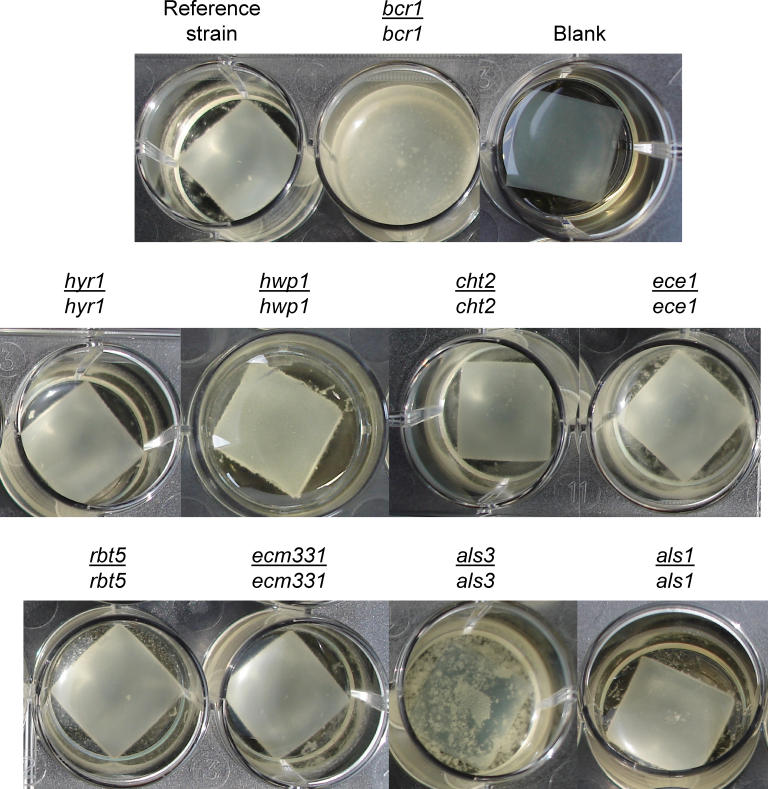
Biofilm Formation In Vitro by Bcr1 Target Gene Mutants Strains were grown in our standard biofilm assay and photographed after 60 h. Relevant genotypes are given above each panel and include DAY286 (reference strain), CJN459 (*bcr1*/*bcr1*), FJS2 (*hyr1*/*hyr1*), CAH7-1A1E2 (*hwp1*/*hwp1*), FJS5 (*cht2*/*cht2*), FJS6 (*ece1*/*ece1*), FJS8 (*rbt5*/*rbt5*), FJS10 (*ecm331*/*ecm331*), CAYF178U (*als3*/*als3*), and CAYC2YF1U (*als1*/*als1*). Turbid medium with all cells free-floating in the medium rather than attached to the silicone substrate indicates a biofilm-negative phenotype; clear medium with the silicone substrate completely covered with cells indicates a biofilm-positive phenotype. An uninoculated control is shown in the panel labeled “Blank.”

**Figure 5:**
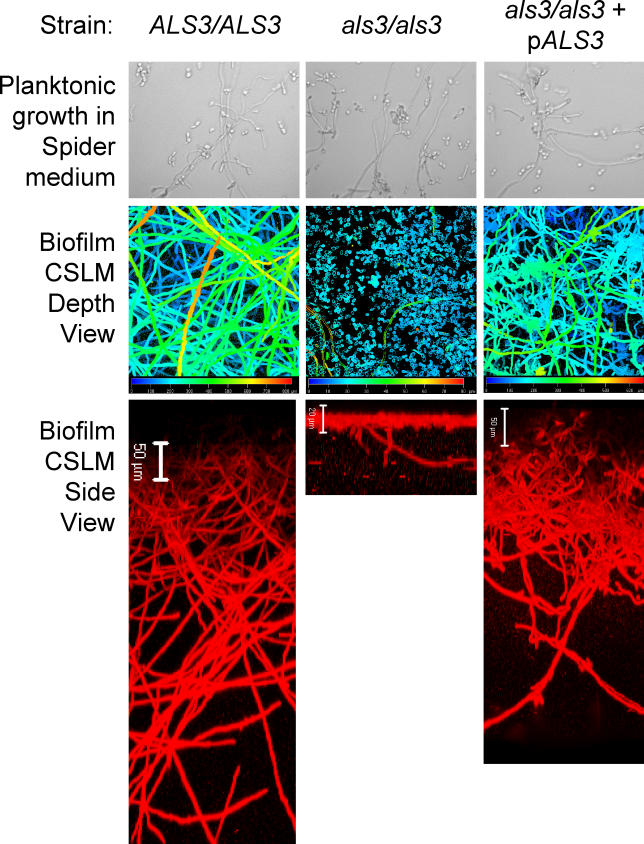
In Vitro Filamentation and Biofilm Formation by the *als3/als3* Mutant Cells were grown in free-living (planktonic) cultures in Spider medium; filamentation was examined by phase contrast microscopy at ×400 magnification (top panels). Biofilms were grown under standard conditions in Spider medium, and stained with concanavalin A conjugate for CSLM visualization. Artificially colored CSLM depth views, in which blue color represents cells closest to the silicone and red color represents cells farthest from the silicone, are shown in middle panels. For the depth views of reference strain CAI4-URA3 (*ALS3*/*ALS3*), blue = 0 μm and red = 800 μm; CAYF178U (*als3*/*als3*), blue = 0 μm, red = 80 μm; CAQTP178U (*als3*/*als3* + p*ALS3*), blue = 0 μm, red = 600 μm. CSLM side views are shown in lower panels. For the side views, the scale bars represent 50 μm for CAI4-URA3 (*ALS3*/*ALS3*) and CAQTP178U (*als3*/*als3* + p*ALS3*); and 20 μm for CAYF178U (*als3*/*als3*).

To test the roles of Bcr1 target genes in biofilm formation, we carried out biofilm formation assays with mutants defective in each gene. We observed no significant biofilm defect in *hyr1/hyr1* (*p* = 0.463)*, ece1/ece1* (*p* = 0.850)*, cht2/cht2* (*p* = 0.909)*,* or *rbt5/rbt5* (*p* = 0.323) mutant strains versus the reference strain (Figure 2, Strain Set B; Figure 4). The *als1*/*als1* and *hwp1/hwp1* mutants also produced substantial biofilms (Figure 4), although the biofilms often sloughed off the substrate. Biofilm biomass determinations further indicated that the *hwp1/hwp1* mutant has a partial biofilm defect compared to the reference strain (Figure 2, Strain Set B, *p* = 0.022). In contrast to the *als1/als1* and *hwp1/hwp1* strains, the *als3/als3* mutant displayed a severe defect in biofilm formation compared to the reference strain (Figure 2, Strain Set B; Figure 4; *p* = 0.005), and introduction of a single wild-type *ALS3* allele rescued the defect substantially (Figure 2, Strain Set B; *p* = 0.005). Confocal scanning laser microscopy (CSLM) imaging revealed that the *als3/als3* mutant formed a rudimentary biofilm of 20 μm in depth, while the wild-type and *als3/als3* + p*ALS3* complemented strains produced biofilms of over 200 μm in depth (Figure 5). CSLM depth images showed that the rudimentary *als3/als3* mutant biofilm was comprised mainly of yeast cells, with few hyphae, whereas the biofilms of the wild-type and *als3/als3* + p*ALS3* complemented strains included abundant hyphae (Figure 5). It should be noted that the *als3*/*als3* mutant is not defective in hyphal formation as it forms normal hyphae when assayed under hyphal inducing conditions (Figure 5). Hyphae are also apparent among the cells in the surrounding medium of an *als3/als3* mutant biofilm (unpublished data). These findings argue that Als3 has a major role in biofilm formation and suggest that reduced expression of *ALS3* in the *bcr1/bcr1* mutant may account for its biofilm defect.

**Figure 6:**
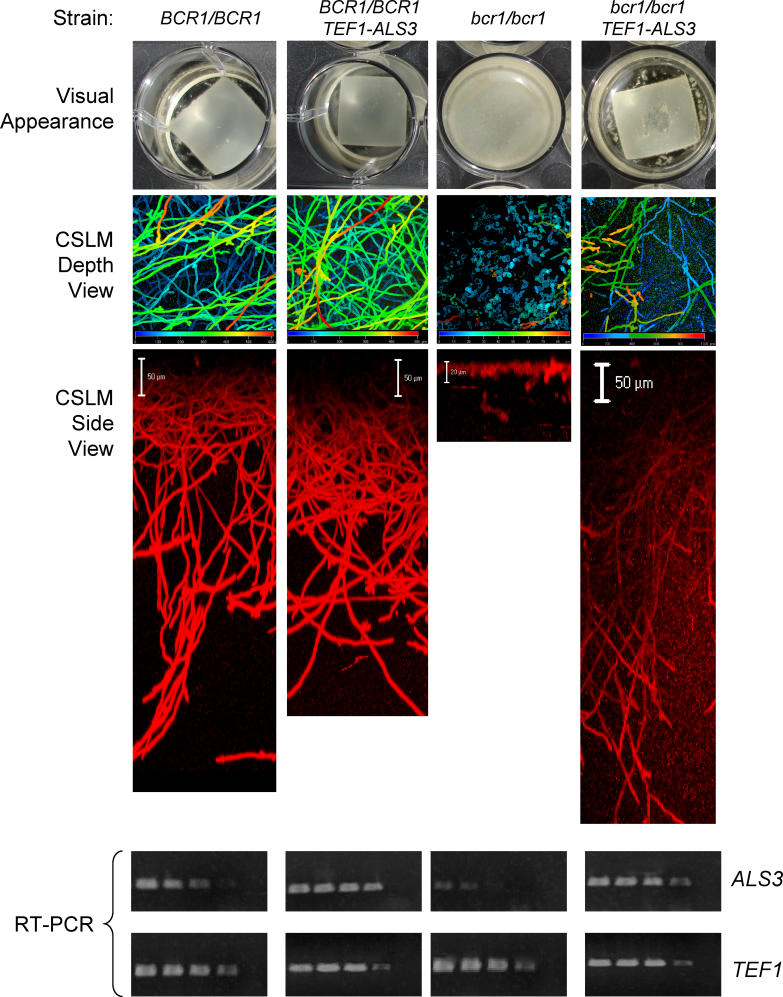
Overexpression of *ALS3* in the *bcr1*/*bcr1* Mutant Restores Substantial Biofilm Formation In Vitro Biofilms were grown under standard conditions and stained with concanavalin A conjugate for CSLM visualization. The top panels show the visual appearance. The next set of panels show depth views, in which blue color represents cells closest to the silicone and red color represents cells farthest from the silicone. The next set of panels show side views. For the depth views of reference strain DAY185 (*BCR1*/*BCR1*), blue = 0 μm and red = 600 μm; CJN1149 (*BCR1*/*BCR1* + *TEF1-ALS3*), blue = 0 μm and red = 500 μm; CJN702 (*bcr1*/*bcr1*), blue = 0 μm and red = 80 μm; CJN1153 (*bcr1*/*bcr1* + *TEF1-ALS3*), blue = 0 μm and red = 180 μm. For the side views, the scale bars represent 50 μm for DAY185 (*BCR1*/*BCR1*), CJN1149 (*BCR1*/*BCR1* + *TEF1-ALS3*), and CJN1153 (*bcr1*/*bcr1* + *TEF1-ALS3*); and 20 μm for CJN702 (*bcr1*/*bcr1*). The next set of panels show RT-PCR analysis of *ALS3* expression of the indicated strains with successive 2-fold dilutions of cDNA from left to right. The bottom panels show RT-PCR of control *TEF1* transcript levels.

If reduced expression of *ALS3* is the cause of the *bcr1/bcr1* mutant biofilm defect, then increased expression of *ALS3* in a *bcr1/bcr1* mutant background should promote biofilm formation. To test this prediction, we introduced the *TEF1* promoter adjacent to the native *ALS3* coding region to create a *TEF1-ALS3* allele, permitting Bcr1-independent *ALS3* expression. RT-PCR measurement of *ALS3* RNA levels confirmed that the *TEF1-ALS3* allele permits expression of *ALS3* in both *BCR1/BCR1* and *bcr1/bcr1* backgrounds (Figure 6). In the wild-type reference strain background, *TEF1-ALS3* had no obvious effect on biofilm formation (Figure 6, top row). In the *bcr1/bcr1* mutant background, *TEF1-ALS3* improved biofilm formation substantially (Figure 2; Figure 6, top row; *p* = 0.002). These observations indicate that increased *ALS3* expression in the *bcr1/bcr1* mutant promotes significant biofilm formation ability.

We used CSLM imaging to examine the structure of biofilms that resulted from increased *ALS3* expression. The *TEF1-ALS3* allele did not alter biofilm structure in the otherwise wild-type background (Figure 6, CSLM depth and side views): biofilm depth was about 400 μm; little staining occurred in the basal region; and hyphal staining was prominent. The *bcr1/bcr1* strain produced a thin rudimentary biofilm comprised largely of yeast form cells, as expected [14]. The *bcr1/bcr1 TEF1-ALS3* strain produced a substantial biofilm that included a basal poorly stained region (Figure 6), similar in appearance to those of the wild-type strain (Figure 6) and complemented *bcr1/bcr1* mutant [14]. Thus, increased *ALS3* expression permits at least partial rescue of the *bcr1*/*bcr1* mutant defect in biofilm formation.

### Bcr1 Function in Biofilm Formation In Vivo

**Figure 7:**
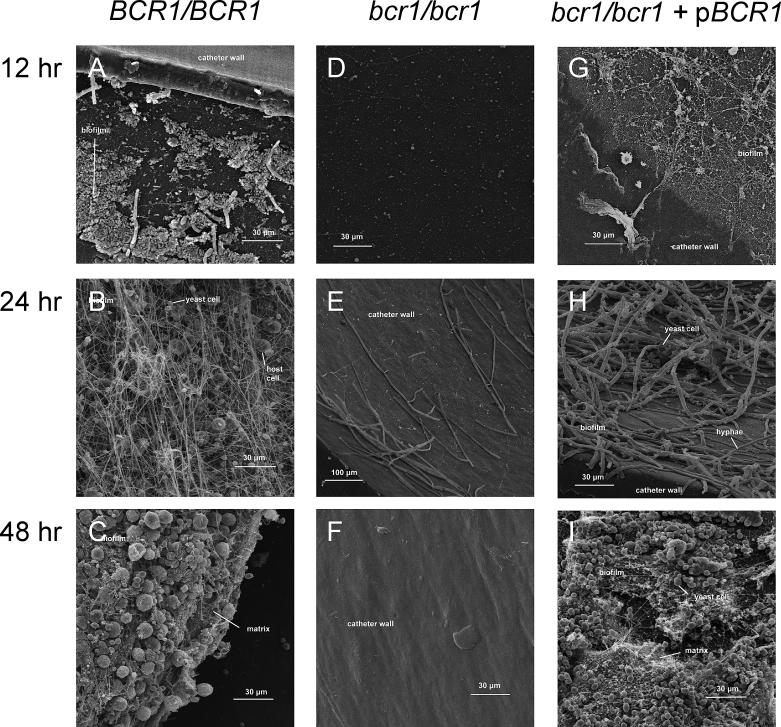
Bcr1 Requirement for Biofilm Formation In Vivo Central venous catheters were introduced into rats, inoculated with C. albicans strain DAY185 (*BCR1/BCR1*) (A–C), CJN 702 (*bcr1/bcr1*) (D–F), or CJN698 (*bcr1/bcr1* + p*BCR1*) (G–I) and then flushed and incubated [18]. Catheters were the removed and their contents visualized by scanning electron microscopy after 12 h (A, D, G), 24 h (B, E, H), and 48 h (C, F, I).

In order to determine whether Bcr1 may have a role in biofilm formation in vivo, we turned to a rat venous catheter model [18]. Implanted catheters were allowed to stabilize for 24 h and were then inoculated with wild-type, *bcr1/bcr1* mutant, or *bcr1/bcr1 +* p*BCR1* complemented strains. Biofilm formation was visualized after 12, 24, and 48 h by scanning electron microscopy of the intraluminal catheter surface (Figure 7). The wild-type and *bcr1/bcr1 +* p*BCR1* complemented strains initiated biofilm formation by 12 h and yielded extensive adherent populations by 24 h (Figure 7A, 7B, 7G, and 7H). Both strains produced mature biofilms by 48 h that included abundant matrix material (Figure 7C and 7I), as previously reported for strain K1 [18]. In contrast, the *bcr1/bcr1* mutant yielded few adherent cells at 12 and 24 h (Figure 7D and 7E), and the catheter surface was devoid of biofilm material after 48 h (Figure 7F). Despite the dramatic differences in biofilm formation ability, the three strains grew comparably in a mouse disseminated infection model; median mouse survival time was 13 d after inoculation with the wild-type strain and 10 d after inoculation with either the *bcr1/bcr1* mutant or *bcr1/bcr1 +* p*BCR1* complemented strains. Based on this evidence, Bcr1 is not required for growth in vivo under non–biofilm-forming conditions but is required for biofilm formation in vivo.

### Als3 Function in Biofilm Formation In Vivo

**Figure 8:**
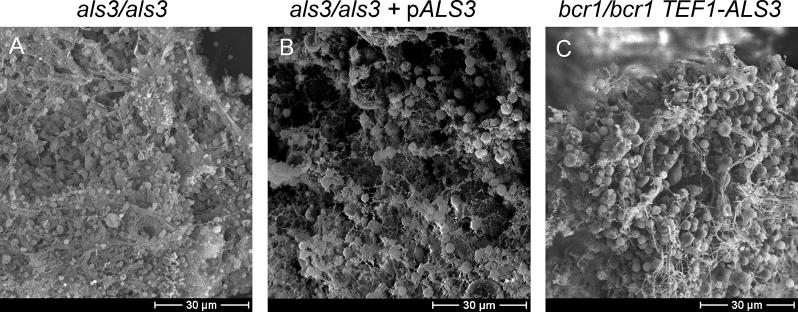
Role of Als3 in Biofilm Formation In Vivo Central venous catheters were introduced into rats, inoculated with C. albicans strains CAYF178U (*als3*/*als3*) (A), CAQTP178U (*als3*/*als3* + p*ALS3*) (B), or CJN1153 (*bcr1*/*bcr1* + *TEF1*-*ALS3*) (C), and then flushed and incubated for 24 h [18]. Catheters were subsequently removed and their contents visualized by scanning electron microscopy.

Our observations above indicate that Als3 is a key mediator of Bcr1-dependent biofilm formation in vitro. To verify that these findings extend to in vivo biofilm formation, we compared *als3/als3* mutant and *als3/als3* + p*ALS3* complemented strains in the rat venous catheter model. Both strains formed extensive biofilms within 24 h (Figure 8A and 8B). Therefore, Als3 is not absolutely required for biofilm formation in vivo.

To determine whether Als3 may contribute to biofilm formation in vivo, we tested the ability of the *TEF1-ALS3* expression construct to rescue the *bcr1/bcr1* mutant biofilm defect. The *bcr1/bcr1 TEF1-ALS3* strain produced an extensive biofilm containing both cells and matrix material (Figure 8C). This biofilm, formed after 24 h, was similar in overall appearance to that formed by the *BCR1/BCR1* control strains (Figures 7B and 8B). *TEF1-ALS3* expression thus rescues biofilm formation in a *bcr1/bcr1* background (compare Figures 7E and 8C). These findings support the model that *ALS3* is a critical Bcr1 target gene that functions in biofilm formation in vivo.

### Overexpression Assays of Bcr1 Target Gene Function In Vitro

Our in vivo assays suggest that Als3 may be one of several Bcr1 targets that contribute to biofilm formation. The analysis of insertion and deletion mutant strains above pointed toward Als1 and Hwp1 as additional candidate functional targets, although their biofilm defects were mild: biofilm biomass was reduced only slightly (Figure 2), and CSLM visualization revealed no qualitative defects (unpublished data). Thus, we turned to an alternative functional analysis strategy, gene overexpression, which has recently been applied with considerable success on a genome-wide scale in Saccharomyces cerevisiae [20]. Gene overexpression is particularly useful in identifying functions among partially redundant genes, the situation that we postulate to exist here.

To determine if increased expression of Bcr1-activated target genes, other than *ALS3*, may rescue the biofilm defect of the *bcr1*/*bcr1* mutant, we created genomic fusions of the *TEF1* promoter to the *CHT2*, *HYR1*, *RBT5*, *ALS1*, *HWP1*, and *ECE1* coding regions. The *TEF1-ALS1*, *TEF1-HWP1*, and *TEF1-ECE1* alleles improved biofilm formation ability considerably (*p* < 0.004 for all comparisons to *bcr1/bcr1*), although not to the extent of *TEF1-ALS3* (Figure 2, Strain Set C; *p* < 0.006 for all comparisons to *bcr1/bcr1 TEF1-ALS3*). These same *TEF1* promoter fusion alleles did not augment biofilm formation in the *BCR1/BCR1* background (unpublished data). These results indicate that Als1, Hwp1, and Ece1 may act in addition to Als3 to contribute to biofilm formation.

## Discussion

**Figure 9:**
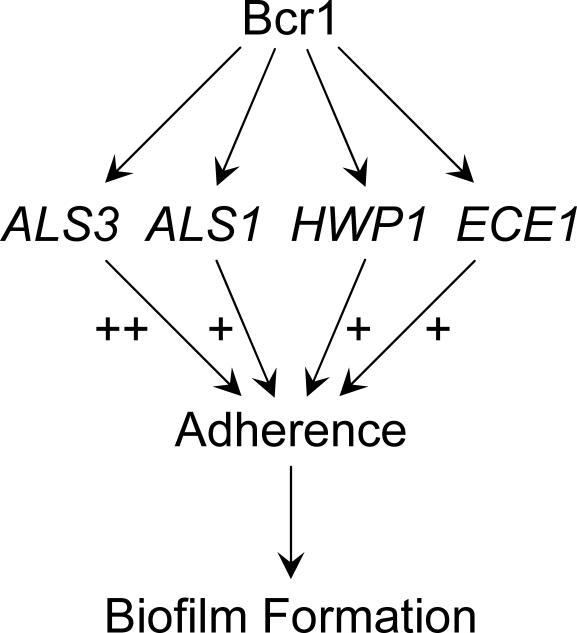
Role of Bcr1 Target Genes in Biofilm Formation Bcr1 is required for full expression of adhesins Als3, Als1, and Hwp1 and of novel protein Ece1. Gene mutation and overexpression analyses together prove that Als3 is necessary and sufficient among Bcr1 targets for biofilm formation in vitro. Overexpression analysis indicates that Als1, Hwp1, and Ece1 can also restore biofilm formation in the absence of Bcr1 in vitro. The fact that overexpression suppressors Als3, Als1, and Hwp1 are all known adhesins indicates that adherence is the property through which Bcr1 governs biofilm formation. Bcr1 is required for biofilm formation in vivo, and overexpression of Als3 permits biofilm formation in the absence of Bcr1 in vivo. Thus, Bcr1-dependent adherence is critical for biofilm formation in vivo and in vitro.

We have recently taken a genetic approach to elucidate the mechanistic basis of C. albicans biofilm formation [14, 15]. A central issue is how in vitro biofilm models are related to biofilm growth in vivo and, thus, to disease. Here we have shown that the transcription factor Bcr1 is required in vivo, as it is in vitro, for biofilm formation. One key target gene under Bcr1 control is *ALS3*, as demonstrated by the rescue of biofilm formation through increased *ALS3* expression in vitro and in vivo. These results argue that Als3-mediated adherence is a key factor in formation of biofilms in vitro and in vivo. However, absence of Als3 causes a biofilm defect only in vitro and not in vivo. One implication from this result is that Bcr1 activates additional biofilm adhesin genes. In support of this model, we find that overexpression of three additional Bcr1 target genes partially restores biofilm formation ability in vitro to a *bcr1/bcr1* mutant. Our findings are summarized in Figure 9. Clearly, the interplay of in vitro and in vivo analyses holds great promise for defining biofilm regulatory mechanisms.

### Relationship of Bcr1 and Hyphal Gene Expression

Our studies here solidify the concept that Bcr1 relays a signal within the hyphal developmental program because an increase in *BCR1* expression leads to increased expression of the hyphal-specific genes *HYR1, HWP1,* and *ALS3* in a hyphal-defective *tec1/tec1* mutant. However, we find that some Bcr1-dependent genes are expressed substantially in the *tec1/tec1* strain, including *RBT5, ECE1,* and *ALS1*, despite the reduced expression of *BCR1.* Two simple explanations can account for this apparent paradox. One possibility is that the 4-fold reduced level of Bcr1 in the *tec1/tec1* mutant is sufficient to activate a subset of target genes. These genes may have the highest-affinity Bcr1 binding sites, or their promoter regions may include binding sites for additional transcription factors that interact cooperatively with Bcr1. A second possibility is that some Bcr1 target genes are subject to a compensatory regulatory mechanism in the *tec1/tec1* background. The latter explanation seems particularly plausible for *RBT5,* which responds to numerous genetic and environmental regulatory signals [21–23]. Identification of the Bcr1 binding site will help to distinguish between these explanations.

One unexpected observation is that *CHT2* expression is both Bcr1 and Tec1 dependent. *CHT2,* which specifies a cell wall chitinase homolog, is expressed at higher levels in yeast cells than hyphal cells under many growth conditions [24, 25]. However, *CHT2* is not exclusively a yeast phase-specific gene. For example, it was found to be coregulated with numerous hyphal-specific genes in a study of pH-regulated gene expression [26], and the *CHT2* transcript has been detected previously in cells induced to form hyphae in Spider medium or serum [21]. Our results indicate that Bcr1 and Tec1 target genes are not restricted to hyphal-specific genes.

### Control of Adherence by Bcr1 during Biofilm Formation In Vitro

The proposal that Bcr1 is a positive regulator of biofilm adherence stems from two prior observations. First, Bcr1 is required for biofilm formation, a process that depends upon both cell-cell and cell-substrate adherence. Second, numerous Bcr1-dependent genes encode proteins that contribute to cell wall or cell surface structure. The in vitro studies reported here include three lines of evidence in support of this proposal. First, expression of Bcr1 in a *tec1/tec1* mutant promotes substantial adherence to a silicone substrate. Second, a deletion of one Bcr1-dependent adhesin gene, *ALS3,* causes a biofilm formation defect similar to that of the *bcr1/bcr1* mutant. Third, the *bcr1/bcr1* biofilm formation defect is fully rescued through increased expression of Als3 and partially rescued through increased expression of two other known adhesins, Als1 and Hwp1. Our results thus indicate that the adhesin expression defect is a major cause of the *bcr1/bcr1* mutant biofilm formation defect.

The *tec1/tec1* mutant has a severe biofilm defect: it grows under our in vitro cultivation conditions as a suspension of yeast cells. Introduction of *TEF1-BCR1* alters that mutant phenotype by promoting growth primarily on the surface of the silicone substrate. The biofilm so formed is unstable in that it disperses into clumps of cells during manipulation, and its biomass is 3-fold less than that of the wild-type and complemented mutant strains. Thus, expression of Bcr1 is not sufficient to promote extensive biofilm formation by yeast cells. However, increased adherence of the *tec1/tec1 TEF1-BCR1* strain, compared to a *tec1/tec1* strain, is readily apparent, thus connecting Bcr1 function to adherence.

The finding that the Bcr1-dependent adhesin Als3 is required for biofilm formation strengthens this connection. Als3 belongs to a large C. albicans protein family with structural features similar to those of the α mating agglutinin of S. cerevisiae [27, 28]. Direct assays have demonstrated roles for C. albicans Als1, Als3, Als5, and Als6 in adherence to diverse substrates [29], and mutational analysis indicates that Als2 and Als4 are also adhesins [30]. The two Bcr1-dependent family members, Als1 and Als3, have highly related sequences throughout their N-terminal domains, the region implicated in substrate binding. This close relationship is reflected by their similar substrate binding properties [29]. Our studies here also support a close functional relationship between Als1 and Als3, because overexpression of either adhesin in a *bcr1/bcr1* background restores biofilm formation to a measurable extent. There are some functional distinctions between Als1 and Als3, because Als3 is required for biofilm formation under our in vitro assay conditions, while Als1 is not. Similarly, *TEF1-ALS3* is more efficient than *TEF1-ALS1* in suppression of the *bcr1/bcr1* biofilm defect. Nonetheless, the clear connection between Bcr1, Als1, and Als3 argues that a major functional role of Bcr1 is to promote adherence.

One unexpected conclusion from our findings is that Hwp1 contributes to biofilm formation in vitro. Hwp1 is a well-characterized hyphal adhesin that serves as a substrate for mammalian transglutaminases, thus mediating covalent attachment of C. albicans to host cells [28, 31]. It has not previously been shown to mediate interactions between C. albicans cells, and the transglutaminases that modify Hwp1 are of mammalian origin [31]. Our observations suggest that Hwp1 can mediate C. albicans cell-cell interactions, and that it does so in the absence of transglutaminase activity. An interesting implication is that Hwp1 may contribute to adherence between mating partners, thus explaining its up-regulation by mating factor [32, 33].

Our findings also implicate Ece1 in adhesion, thus providing the first functional insight into this protein. *ECE1* was discovered as a hyphal-induced gene and was among the first C. albicans genes disrupted with the Ura-blaster method [34, 35]. However, the *ece1/ece1* mutant has no apparent phenotypic defect [35]. The idea that Ece1 functions in adhesion is suggested by our observation that its overexpression restores biofilm formation to a *bcr1/bcr1* mutant. *ECE1,* like *HWP1,* is induced by mating pheromone [33], another possible connection between Ece1 and adherence. Ece1 does not resemble an adhesin: it is comprised of novel 34-residue repeats that surround a possible transmembrane domain [35]. Although its mechanism of action is uncertain, an interesting possibility is that Ece1 promotes surface exposure of adhesins.

### Genetic Control of Biofilm Formation In Vivo

Biofilm formation in vivo is considerably more complex than in vitro and involves dynamic interactions with many host proteins, cells, and environmental factors. These differences raise the question of whether the major genetic factors operative in vitro play a commensurate role in vivo. We have addressed this issue for two gene products: Bcr1 and Als3. Although the experimental outcomes were different in detail, they argue that both proteins have significant roles in vivo.

The significance of Bcr1 is clearest: it is required in vivo for biofilm formation but not for growth. The fact that the mutant leaves catheter surfaces essentially clear of material suggests that there is a defect in early events of biofilm formation in vivo, much as observed in vitro. The defects under the two circumstances, however, are slightly different: a thin layer of *bcr1/bcr1* mutant cells is associated with the substrate transiently in vivo but stably in vitro. It is possible that the few substrate-bound cells that appear early in vivo may be destroyed later by host defenses. An alternative possibility is that larger cell masses are dislodged efficiently by blood flow if their adherence is compromised by the *bcr1* defect. In either case, it is clear that Bcr1 governs a mechanism that contributes to biofilm formation in vivo.

The potency of *TEF1-ALS3* as a suppressor of the *bcr1/bcr1* defect argues that Als3 also has a critical role in biofilm formation in vivo. How can that observation be reconciled with the fact that an *als3/als3* null mutant has no biofilm defect in vivo? One simple model is that additional adhesins can partially compensate for the absence of Als3 in vivo but not in vitro. Our overexpression studies implicate Als1 and Hwp1 as candidate compensatory adhesins, in keeping with this model. The distinction between the in vivo and in vitro situations may reflect a higher level of expression of the compensatory adhesins in vivo than in vitro. A second possibility is that host constituents, for example serum components, may contribute to adherence. Thus, the same low level of surface adhesin activity may support biofilm formation in vivo but not in vitro.

The restoration of biofilm formation through *ALS3* overexpression in the *bcr1/bcr1* mutant both in vitro and in vivo indicates that Bcr1 governs one main function relevant for biofilm formation: adherence. Although transcription factor mutants are useful for definition of functionally related genes, the mechanistic basis for their phenotypic defects can be complex because of the extent of their gene expression defects. Moreover, functional overlap among targets can obscure loss-of-function target gene mutant phenotypes. Our results here illustrate the utility of gene overexpression for identification of critical target genes that govern a complex process.

## Materials and Methods

### Media.

C. albicans strains were grown at 30 °C in either YPD (2% Bacto Peptone, 2% dextrose,1% yeast extract) for Ura+ strains or in YPD+uri (2% Bacto Peptone, 2% dextrose,1% yeast extract, and 80 μg/ml uridine) for Ura− strains. C. albicans transformants were selected for on synthetic medium (2% dextrose, 6.7% YNB with ammonium sulfate, and auxotrophic supplements) or on YPD+clonNAT (2% Bacto Peptone, 2% dextrose,1% yeast extract, and 400 μg/ml clonNAT [WERNER BioAgents, Jena, Germany]) for Nat+ strains. For biofilm growth, strains were grown at 37 °C in Spider medium [36]. Assays for hyphal induction of the *tec1*/*tec1* mutant (+ vector) (CJN1052), the *tec1*/*tec1* mutant overexpressing *BCR1* (CJN1035), the reference strain (+ vector) (CJN1015), the reference strain (DAY185), the *als3*/*als3* mutant (CAYF178U), and the *als3*/*als3* +p*ALS3* complemented strain (CAQTP178U) were also done at 37 °C in Spider medium.

### Plasmid and C. albicans strain construction.

All strains used in this study are listed in Table 1. All strains are derived from BWP17 (*ura3*Δ::λ*imm434*/*ura3*Δ::λ*imm434 arg4*::*hisG*/*arg4*::*hisG his1*::*hisG*/*his1*::*hisG*) [37] except for the following CAI4 derivatives [34]: CAI4-URA3 [38], CAYC2YF1U, the *als1*/*als1* mutant strain [39], and CAH7-1A1E2 [28], the *hwp1*/*hwp1* mutant strain. Construction of the *bcr1*/*bcr1* insertion mutant strain, CJN459; the *tec1*/*tec1* insertion mutant strain, CJN308; the *bcr1*/*bcr1* deletion mutant strain, CJN702, and its complemented strain, CJN698, was described previously [14].

**Table 1:**
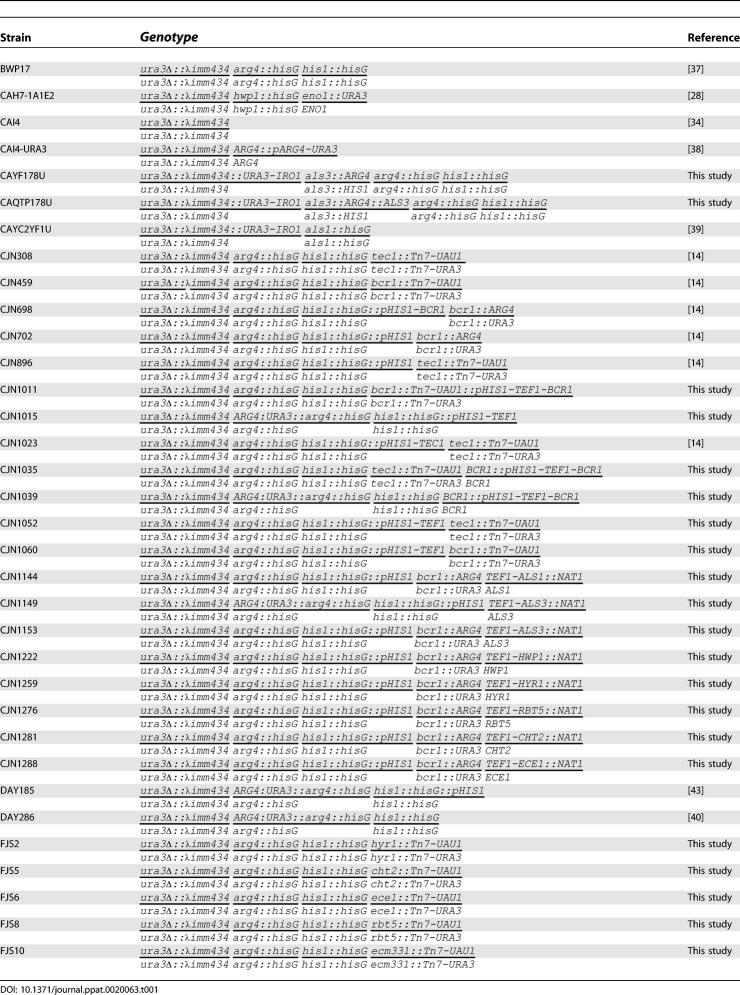
C. albicans Strains Used in This Study

For construction of the insertion mutant strains for Bcr1 target genes, we took advantage of a *Tn7-UAU1* plasmid insertion mutant library containing our genes of interest, made by The Institute for Genome Research (TIGR). Each TIGR plasmid containing the *orf::Tn7-UAU1* segment for *orf19.4975* (*HYR1*), *orf19.3895* (*CHT2*), *orf19.3374* (*ECE1*), *orf19.5636* (*RBT5*), and *orf19.4255* (*ECM331*) was released by NotI digestion and then transformed into strain BWP17 using standard C. albicans transformation protocols described previously [40], except that C. albicans cells were heat shocked at 44 °C for 20 min, which increased efficiency, instead of the standard 42 °C for 1 h. The Arg+ heterozygous transformants were then used to obtain Arg+ Ura+ homozygous insertion mutant strains FJS2 (*hyr1*/*hyr1*), FJS5 (*cht2*/*cht2*), FJS6 (*ece1*/*ece1*), FJS8 (*rbt5*/*rbt5*), and FJS10 (*ecm331*/*ecm331*) using methods described previously [40]. These homozygous insertion mutants were then screened by colony PCR to ensure absence of the wild-type allele. We used strain DAY286 (Arg+ Ura+ His−) [40] as a reference strain for these mutants.

For construction of the *TEF1-BCR1* overexpression plasmid pCJN491, PCR was done using primers OE723-ATG (5′-ATGTCAGGGACATCACAAGTACTTCA-3′) and OE723-908 (5′-AATAATAGTTTCCCAATTGAAAAAAGAGAGGAC-3′) to generate a 2,723-bp fragment beginning from the ATG of the *BCR1* ORF (*orf19.8342*) to 500 bp downstream of the stop codon. This fragment was inserted into the pGEMT-Easy vector (Promega, Madison, Wisconsin, United States) and then digested with EcoRI and SpeI (releasing a 1,650-bp fragment containing the larger portion of the *BCR1* ORF including the start codon and 1,650 bp downstream of the start codon), and cloned into an EcoRI- and SpeI-linearized vector pTEF1 [15], to yield plasmid pCJN491 in the correct orientation. pTEF1 [15] is a vector that harbors the constitutively active *TEF1* promoter that is derived from pDDB78, a *HIS1* vector [41]. A unique SbfI site lying within the 1,650-bp portion of *BCR1* was used to direct integration of the plasmid to the natural *BCR1* locus via SbfI digestion. The *TEF1-BCR1* overexpression C. albicans strains CJN1011, CJN1035, and CJN1039 were constructed by transforming CJN459 (a His− *bcr1*/*bcr1* insertion mutant), CJN308 (a His− *tec1*/*tec1* insertion mutant), and DAY286 (a His− reference strain), respectively, with SbfI-linearized pCJN491 to generate His+ strains overexpressing *BCR1*. The *TEF1* vector alone C. albicans strains CJN1060, CJN1052, and CJN1015 were constructed by transforming CJN459, CJN308, and DAY286, respectively, with NruI-linearized pTEF1 to generate His+ strains with the vector alone.

The *NAT1-TEF1* overexpression plasmid pCJN498 was generated as follows. PCR was done using primers AgNat1F (5′-ATCAAGCTTGCCTCGTCC-3′) and AgNat1R (5′-GCGTTAGTATCGAATCGACAG-3′) with the template plasmid pJK799 [42] to generate a 1,220-bp fragment amplifying the *Ashbya gossypii TEF1* promoter next to the *C. albicans NAT1* ORF and followed by the *A. gossypii TEF1* terminator. The use of A. gossypii sequences instead of C. albicans sequences in pJK799 surrounding the *NAT1* ORF prevents misintegration of the construct [42]. This fragment was inserted into the pGEMT-Easy vector (Promega) in the correct orientation to create plasmid pCJN495. PCR was done using primers TEF1-SpeIF (5′-AAACTAGTGCATCTAAACATCAATTGAC-3′) and TEF1-Nde1R (5′-GATTGATCATATGTATATAAAATGTATACTTAG-3′) to generate an 800-bp product containing the *C. albicans TEF1* promoter with added NdeI and SpeI restriction sites upstream and downstream of the promoter, respectively. This PCR fragment was digested with NdeI and SpeI and ligated into NdeI- and SpeI-digested plasmid pCJN495 to create pCJN498 containing the *A. gossypii TEF1* promoter next to the *C. albicans NAT1* ORF, followed by the *A. gossypii TEF1* terminator, followed by the *C. albicans TEF1* promoter in the correct orientation.

The *TEF1-ALS3* overexpression C. albicans strains CJN1149 and CJN1153 were constructed by transforming DAY185 (a His+ reference strain) [43] and CJN702 (a His+ *bcr1*/*bcr1* deletion mutant), respectively, using PCR products from template plasmid pCJN498 and primers ALS3-F-OE-Ag-NAT-Ag-TEF1p (5′-AGCCAAACAATCCGAAGCAACGTAAAGTACGATATCAAAGAATCATAACTTTGCTTTCTATTTGATAACCCGCCTCAAATCAAGATTGGGAGGTTAACAATCAAGCTTGCCTCGTCCCC-3′) and ALS3-R-OE-Ag-NAT-Ag-TEF1p (5′-TAGACCAAGTCAATGAATTAAAACTGTTGAAAACACCAGTGATTGTCTTTGCAGTCGCAACCGACAAATATATGAGTAACAATGTATATTGTTGTAGCATTATAAAATGTATACTTAGAA-3′). These primers amplify the entire *A. gossypii TEF1* promoter, the *C. albicans NAT1* ORF, the *A. gossypii TEF1* terminator, and the *C. albicans TEF1* promoter with 100 bp of hanging homology to 500 bp upstream into the promoter of *ALS3* for the forward primer and 100 bp of hanging homology from exactly the start codon of the *ALS3* ORF. The homology in these primers allows for homologous recombination of the entire cassette directly upstream of the *ALS3* natural locus so that *ALS3* can be overexpressed with the *TEF1* promoter instead of its natural promoter. The transformation into C. albicans strains was done as described above except an additional 5-h recovery step in YPD at 30 °C was done after the cells were heat shocked at 44 °C for 20 min in order to allow for *NAT1* expression. The cells were then plated onto YPD + 400 μg/ml clonNat plates for 2 d at 30 °C to select for Nat+ transformants, and transformants were checked by colony PCR. We used strain DAY185 (Arg+ Ura+ His+) [43] as a reference strain for these strains.

The *als3*Δ/*als3*Δ mutant, CAYF178U, was constructed from strain BWP17. The two alleles of *ALS3* were serially disrupted using the markers *HIS1* and *ARG4*. The disruption cassettes were amplified with the following primers: ALS3-5DR (5′-CCTCATTACACCAACCATACAACTTTGTGGTCTACAACTTGGGTTATTGAAACAAAAACAGTTTTCCCAGTCACGACGTT-3′) and ALS3-3DR (5′-GGTTGATTCAGCAGTAGTAGTAACAGTAGTAGTTTCATCAGCACTAGAAGAAATGATAGGTGTGGAATTGTGAGCGGATA-3′). The disruption of *ALS3* was verified by PCR using the following primers: 3Confirm-1 (5′-ATGACACCATGTCAAGTTCAGA-3′) and 3Confirm-2 (5′-GTTGGTTGTTCAATGACACTGG-3′). To complement the *als3*Δ*/als3*Δ mutant with a wild-type copy of *ALS3,* a full–length version of *ALS3* was digested from pGEMT with PvuI and SphI [29], and then subcloned into pDS10 at the SphI site [44]. The construct was linearized with Bsp1407I and integrated into the *ALS3* locus of the *als3*Δ/*als3*Δ Ura− strain, selecting Ura+. Excision of the *URA3-dpl200* marker was then selected by plating on 5-FOA medium. *ALS3* complementation was confirmed by PCR using primers 5′-TGAAGCAGCCTTTAGTGGCCT-3′ and 5′-AGAAGTGGAAGCAGCTGTGGA-3′.

*URA3* and the adjacent *IRO1* locus was restored in the *als1*Δ/*als1*Δ strain [39], *als3*Δ/*als3*Δ, and *als3*Δ/*als3*Δ::*ALS3* strains as follows. Ura− derivatives of these mutants were selected by plating on synthetic media containing 5-FOA and uridine. A 3.9-kb *URA3-IRO1* fragment was released from pBSK-URA3 by NotI/PstI digestion and used to transform the Ura− strains [44]. The restoration of *URA3* to its native locus was confirmed by PCR using the primers 5′-TGCTGGTTGGAAT GCTTATTTG-3′ and 5′-TGCAAATTCTGCTACTGGAGTT-3′.

### In vitro biofilm growth conditions.

For in vitro biofilm growth assays, strains were grown in YPD overnight at 30 °C, diluted to an OD_600_ = 0.5 in 2 ml of Spider medium (with auxotrophic supplements), and added to a sterile 12-well plate with a prepared silicone square (1.5 × 1.5 cm cut from Cardiovascular Instrument silicone sheets [Wakefiled, Massachusetts, United States]). The silicone square was previously treated with bovine serum (B-9433; Sigma, St. Louis, Missouri, United States) overnight and washed with PBS in order to prepare it for the biofilm assay. The inoculated plate was incubated at 37 °C for 90 min at 150 rpm agitation for initial adhesion of cells. To remove unadhered cells, the squares were washed with 2 ml of PBS, and the squares were moved to a fresh 12-well plate containing 2 ml of fresh Spider medium. This plate was incubated at 37 °C for an additional 60 h at 150-rpm agitation to allow for biofilm formation.

### Microscopic visualization of in vitro biofilms.

For the in vitro experiments, biofilms were observed visually and by CSLM. For in vitro CSLM imaging, biofilms were stained with 25 μg/ml concanavalin A Alexa Fluor 594 conjugate (C-11253; Molecular Probes, Eugene, Oregon, United States) for 1 h in the dark at 37 °C with 150 rpm agitation. CSLM was performed with an upright Zeiss Axioskop2 FS MOT LSM 510 multiphoton microscope using a Zeiss Achroplan ×40/0.8W objective. In order to visualize concanavalin A conjugate staining, a HeNe1 laser with 543-nm excitation wavelength was used. All in vitro CSLM images were assembled into side and depth views using the Zeiss LSM Image Browser (version 3.2.0.115) software. For all side views, the silicone is located at the top of the image. Depth views are artificially colored images indicating cell depth using a color gradient, where blue represents cells closest to and red represents cells farthest from the silicone substrate.

### RNA isolation and expression analysis.

Overnight cultures were inoculated in 5 ml of YPD at 30 °C. The next day, 100 ml of Spider medium was inoculated with the YPD overnight culture to obtain an OD_600_ = 0.05, and was grown at 37 °C for 12 h (OD_600_ = ≈8). Cells were immediately harvested by vacuum filtration. RNA extraction and Northern analysis were performed as previously described [40]. For RT-PCR analysis for detection of *ALS1* and *ALS3*, 10 μg of total RNA was DNase treated at 37 °C for 1 h, ethanol precipitated, and resuspended in 100 μl of DEPC water. cDNA was synthesized and RT-PCR was done as previously described for *ALS1* and *ALS3* [45] with reverse transcriptase and without reverse transcriptase (as a control).

### In vivo biofilm model.

A rat central venous catheter infection model [18] was selected for in vivo biofilm studies. The catheter diameter was chosen in an attempt to permit blood flow around the extraluminal catheter surface. To mimic material used in patients, polyethylene tubing (inner diameter −0.76 mm, outer diameter 1.52 mm) was chosen. Specific-pathogen-free Sprague-Dawley rats weighing 400 g were used (Harlan Sprague-Dawley, Indianapolis, Indiana, United States). A heparinized (100 U/ml) catheter was surgically inserted into the external jugular vein and advanced to a site above the right atrium (2 cm length). The catheter was secured to the vein and the proximal end tunneled subcutaneously to the midscapular space and externalized through the skin. The catheters were placed 24 h prior to infection to allow a conditioning period for deposition of host protein on the catheter surface. Infection was achieved by intraluminal instillation of 500 μl of C. albicans cells (10^6^ cells/ml). After a dwelling period of 4 h, the catheter volume was withdrawn and the catheter was flushed with heparinized 0.15 M NaCl.

Catheters from two animals were removed at three time points (12, 24, and 48 h) after C. albicans infection to determine biofilm development on the internal surface of the intravascular devices. The distal 2 cm of the catheter was cut from the entire catheter length, and biofilms were imaged using both CSLM and scanning electron microscopy. Scanning electron microscopy was used for architectural investigation of the biofilm process. Catheter segments were washed with 0.1 M phosphate buffer (pH 7.2) and placed in fixative (1% glutaraldehyde and 4% formaldehyde). The samples were then washed with buffer for 5 min and placed in 1% osmium tetroxide for 30 min. The samples were then dehydrated in a series of 10-min ethanol washes (30%, 50%, 70%, 85%, 95%, and 100%). Final desiccation was accomplished by critical point drying (Tousimis, Rockville, Maryland, United States). Specimens were mounted on aluminum stubs and sputter-coated with gold. Samples were imaged in a scanning electron microscope (Hitachi S-5700 or JEOL JSM-6100) in the high-vacuum mode at 10 kV. The images were processed for display using Adobe Photoshop 7.0.1.

### Disseminated murine candidiasis models.

Groups of ten 20-g male Balb/C mice were inoculated via the lateral tail vein with 5 × 10^5^ blastospores with each strain of C. albicans. The mice were monitored three times daily for survival.

### Biofilm dry mass measurements.

For dry mass measurements, each silicone square was weighed prior to inoculation with the strain of interest. Biofilms were grown for 60 h on the silicone square (as described above). The silicone squares containing their respective biofilms were then removed from the wells, dried overnight in a fume hood, and weighed the following day. Total biomass of each biofilm was calculated by subtracting the weight of the silicone prior to biofilm growth from the weight of the silicone after biofilm growth. The average total biomass for each strain was calculated from four independent samples after subtracting the mass of a blank silicone square with no cells added. Statistical significance (*p-*values) was calculated with the Student's two-tailed *t*-test function in Microsoft Excel.

## Supporting Information

Figure S1 Mouse Survival Data Disseminated murine candidiasis assays. Groups of ten 20-g male Balb/C mice were inoculated via the lateral tail vein with 5 × 10^5^ blastospores with each strain of C. albicans. The mice were monitored three times daily for survival.
(44 KB PDF)

### Accession Numbers

Information for the following C. albicans genes can be found at the *Candida* Genome Database (CGD) Web site (http://www.candidagenome.org): *BCR1* (orf19.723), *TEC1* (orf19.5908), *HWP1* (orf19.1321), *ALS3* (orf19.1816), *ALS1* (orf19.5741), *HYR1* (orf19.4975), *CHT2* (orf19.3895), *ECE1* (orf19.3374), *RBT5 (orf19.5636),* and *ECM331* (orf19.4255).

## Abbreviations

CSLM: confocal scanning laser microscopy
