# Supplementary material for: Critical Role of Bcr1-Dependent Adhesins in C. albicans Biofilm Formation In Vitro and In Vivo
Source: PLoS Pathog. 2006 Jul 7;2(7):e63. doi: 10.1371/journal.ppat.0020063 (PMC1487173; doi:10.1371/journal.ppat.0020063)
Supplement: Supplementary file 1 [file ppat.0020063.sg001.pdf]

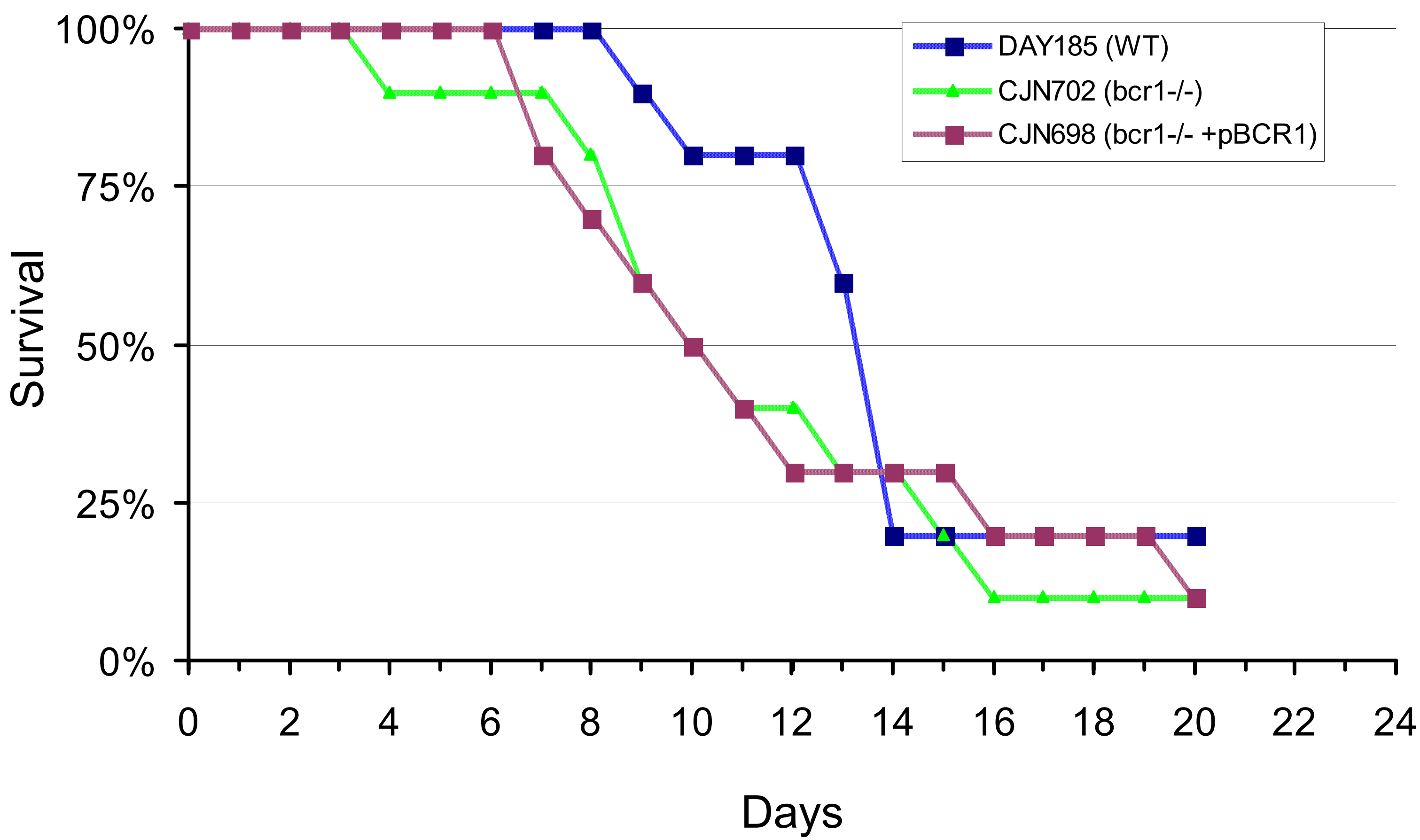

Disseminated murine candidiasis assays. Groups of ten 20 g male Balb/C mice were inoculated via the lateral tail vein with 5 x10^5 blastospores with each strain of *C. albicans*. The mice were monitored three times daily for survival.
